# Lower Extremity Fibro-Adipose Vascular Anomaly: A Post-surgical Rehabilitation Treatment

**DOI:** 10.7759/cureus.62708

**Published:** 2024-06-19

**Authors:** Mafalda Cunha, Filipa Gonçalves, Gabi Almeida, Maria João Azevedo, João Cunha

**Affiliations:** 1 Physical Medicine and Rehabilitation, Hospital Senhora da Oliveira - Guimarães, Guimarães, PRT; 2 Physical Medicine and Rehabilitation, North Rehabilitation Centre, Gaia, PRT; 3 Physical Medicine and Rehabilitation, Hospital de Braga, Braga, PRT

**Keywords:** muscle contracture, vascular malformations, rehabilitation program, gastrocnemius resection, fibro-adipose vascular anomaly

## Abstract

Fibro-adipose vascular anomaly (FAVA) presents diagnostic and therapeutic challenges due to its rarity and overlapping features with other vascular malformations. Predominantly affecting the lower extremities, it manifests with pain and contracture, and surgical resection may be necessary in symptomatic cases. We present a case of a 36-year-old patient with FAVA in the right lower extremity, experiencing persistent symptoms since adolescence. The condition was managed with surgical gastrocnemius resection. Following surgery, the patient underwent a comprehensive rehabilitation program, resulting in significant clinical and functional improvement. This case highlights the importance of tailored interventions in FAVA. The challenges encountered in diagnosing and managing FAVA underscore the necessity for continued research and clinical discourse to improve patient care. Our report emphasizes the significance of collaborative and multidisciplinary care in maximizing functional recovery and quality of life post-gastrocnemius resection, highlighting the importance of optimized rehabilitation programs.

## Introduction

Fibro-adipose vascular anomaly (FAVA) is a rare complex vascular malformation first described by Alomari and colleagues in 2014 [1]. These lesions typically emerge during childhood, with symptoms manifesting in early adolescence. While somatic mutations in the PIK3CA gene are often implicated, diagnosing FAVA poses challenges due to overlapping clinical and imaging features with other vascular malformations and tumors. Predominantly affecting females, patients with FAVA present with a constellation of symptoms, including persistent pain, discomfort, functional impairment, and contracture. FAVA commonly manifests in the lower extremities, particularly the calf muscles. Pain, often accompanied by limited ankle dorsiflexion and calf contractures, is the most prevalent symptom, with additional manifestations such as localized swelling and paresthesia. Characterized histologically by fatty and dense fibrous tissue with venous malformation involvement, FAVA poses diagnostic challenges. Imaging modalities such as MRI and ultrasonography play a crucial role in diagnosis, revealing characteristic features such as a solid fibrofatty intramuscular lesion with low-flow vascular malformations. While non-invasive treatments like sirolimus therapy, cryoablation, and, in less extent, sclerotherapy can provide relief, surgical resection remains the treatment of choice for symptomatic lesions and progressive contractures [1-7].

## Case presentation

A 36-year-old female, employed as an operational assistant, sought medical attention due to FAVA manifestation in her right lower extremity. She had undergone two cesarean sections, used contraceptive medication, and had no reported allergies. Notably, she had a twin sister who was asymptomatic.

**Figure 1:**
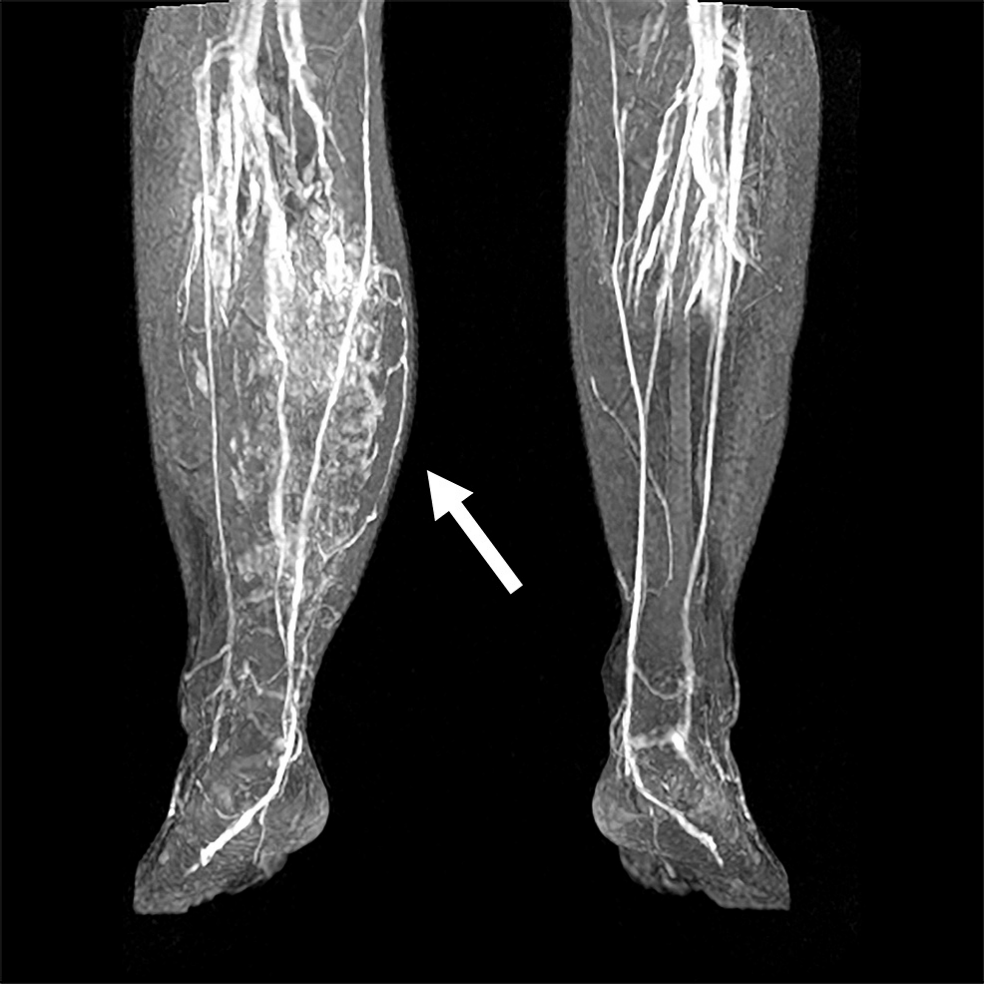
Magnetic resonance imaging illustrating the FAVA lesion within the right gastrocnemius muscle (arrow).

**Figure 2:**
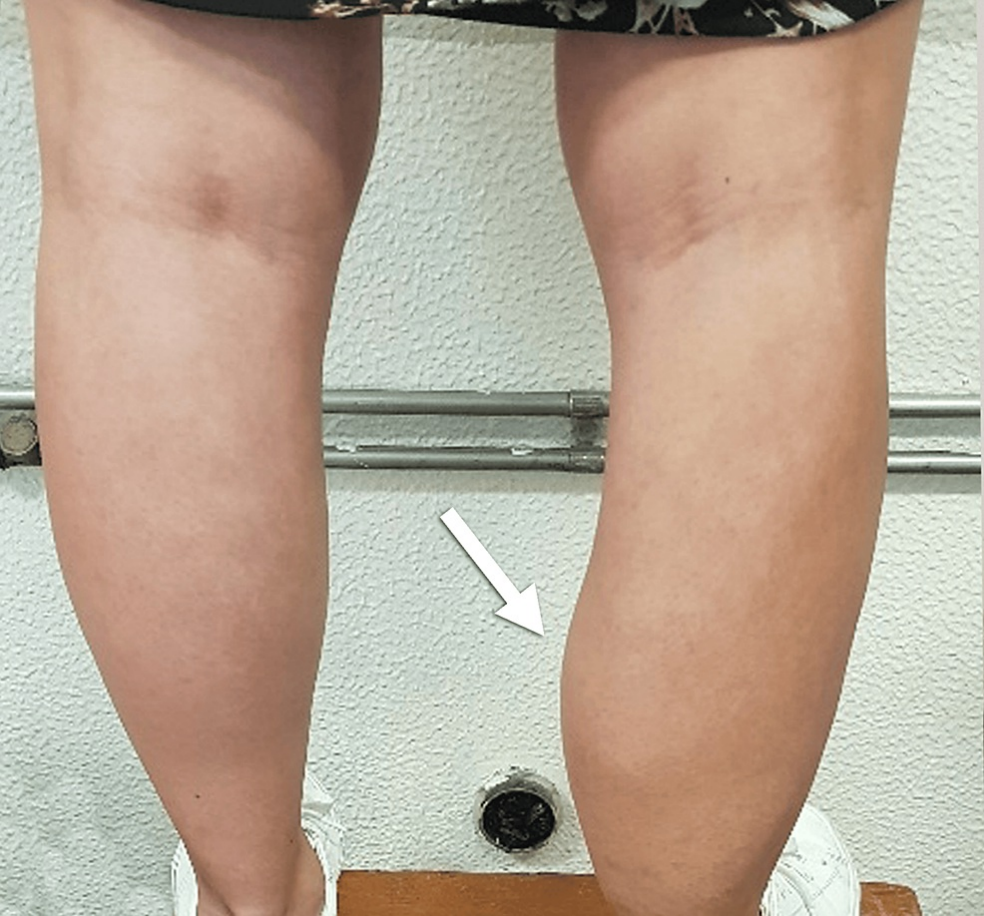
Photograph of the patient's legs before surgery, highlighting the presence of the FAVA lesion in the right leg (arrow).

At the age of 13 years old, she noticed swelling on the inner distal aspect of her right leg, accompanied by night pain. A Doppler ultrasound at that time revealed an unspecified anomaly. Since adolescence, she experienced daily morning moderate pain in the inner distal aspect of her right leg, relieved by walking, with occasional severe and continuous pain flares, particularly after physical activity, leading to sleep disruption. During her pregnancies at 19 and 27 years old, she observed worsening swelling, though less pronounced during the last pregnancy. Since then, she has exhibited leg edema, limited ankle mobility, and difficulties with squatting, jumping, and running. Furthermore, her running pattern differed from that of her peers, and running itself caused her pain afterward. She reported no sensory changes, gait instability, need for walking aids, or difficulties in climbing stairs. She refrained from analgesic medication targeted at this issue and never underwent a rehabilitation program tailored to this problem. Since late adolescence, she has been under vascular surgery consultation, undergoing serial radiological assessments with magnetic resonance imaging (MRI) (Figure 1). About five years ago, she received her diagnosis of FAVA. However, due to the recent growth of the mass (Figure 2), surgical intervention was proposed.

At the age of 36 years old, she underwent surgical excision of the FAVA lesion, which involved the removal of the right medial gastrocnemius muscle and a portion of the lateral gastrocnemius muscle. Following the procedure, she was advised to partially bear weight with the aid of crutches and was referred for a consultation with a physical medicine and rehabilitation (PM&R) specialist. During the PM&R consultation, 10 days post-surgery, suture staples were still present, and significant edema, noticeable plantar flexor shortening, and muscle weakness were observed. Due to pain, she could only bear 20% of her body weight on the affected leg, leading to the postponement of the initiation of her rehabilitation program. By the one-month follow-up, her pain had subsided, and she could walk with crutches. Subsequently, a daily outpatient rehabilitation program was prescribed, comprising cryotherapy, massage, gentle stretching, strengthening exercises, and gait retraining. It is worth noting that her treatment plan was formulated through collaborative discussions among various PM&R specialists, her vascular surgeon, and her physiotherapist.

At the two-month follow-up, she reported being pain-free but experienced fatigue during prolonged walks. There was a notable improvement in ankle joint mobility and muscle strength, although dorsiflexion remained limited (maximum 10º). Consequently, the rehabilitation program progressed to include additional dynamic strengthening and plyometric exercises, as well as progressively challenging gait, stair, and running training.

By the four-month follow-up, the patient contracted a SARS-CoV-2 infection, and experienced a sensation of "tightness" in her leg, along with decreased ankle dorsiflexion and muscle weakness. Dynamometry testing revealed a 20% decrease in muscle strength across all muscle groups compared to the contralateral side. At this time, she was discharged from hospital-based rehabilitation and prescribed a home-based rehabilitation program.

**Figure 3:**
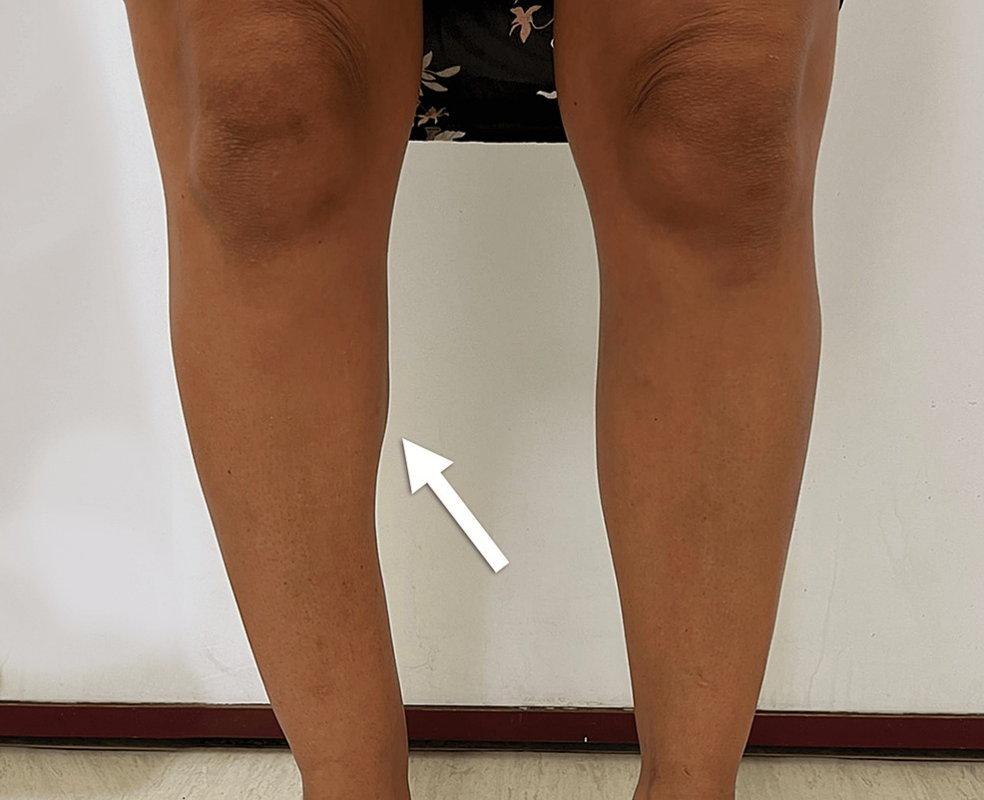
Photograph of the patient's legs post-surgery, following resection of the right medial gastrocnemius muscle (arrow).

At the six-month follow-up (Figure 3), the patient was asymptomatic, although dorsiflexion remained limited (maximum 10º). Muscle strength testing revealed a 20% deficit in gastrocnemius strength compared to the contralateral side, with similar strength observed in all other lower limb muscles, including the soleus muscle. She achieved 60% performance on functional testing (hop test for distance, side hop test, and triple hop test) compared to the contralateral side, leading to her discharge from further rehabilitation.

## Discussion

This report delineates a prototypical case of FAVA, aligning with the limited cohort documented by Alomari and fellow researchers. The patient, a female, initially discerned a painful calf mass at the age of 13. Nonetheless, the elusive nature of the disease comprehension at the time hindered a definitive diagnosis. Preceding surgical intervention, she displayed a spectrum of musculoskeletal manifestations commonly associated with FAVA, including pain exacerbated during physical exertion, calf swelling, contracture, restricted ankle mobility, and functional impairment. These manifestations align with the sparse instances reported in the extant literature on FAVA [1-9].

In our instance, surgical excision of the causative FAVA lesion was executed, mandating substantial resection of the gastrocnemius. Given the absence of established rehabilitation protocols tailored to FAVA cases, we based our intervention on programs designed for gastrocnemius resections for alternative indications [10,11].

Post-comprehensive rehabilitation, the patient persisted with limited dorsiflexion, likely attributable to longstanding joint contracture. Additionally, she evidenced a 20% reduction in gastrocnemius muscle strength compared to the contralateral side, congruent with expectations of post-extensive muscle excision. Moreover, her performance on hop tests notably diminished, achieving only 60% compared to the unaffected side.

These findings can be elucidated within the anatomical and functional disparities between the gastrocnemius and soleus muscles, integral constituents of the triceps surae responsible for ankle plantar flexion [12]. While the predominantly type I fiber composition of the soleus sustains posture, the more balanced fiber distribution in the gastrocnemius facilitates rapid, explosive ankle joint movements [13,14]. Consequently, the functional sequelae of gastrocnemius resection corroborate with our prognostic expectations, particularly evident in compromised hop test performance, a significant outcome. This concurrence parallels observations from other contexts of gastrocnemius resection for varied etiologies, thereby underscoring the broader implications of such procedures [15,16].

To our knowledge, there are no documented cases of rehabilitation post-gastrocnemius resection for FAVA in existing literature. It appears imperative to furnish additional descriptions to optimize the functional outcomes of such patients through interdisciplinary clinical discourse.

With the excision of the malformation, the anticipation of no associated lesions arises. Hence stability of the clinical picture would be presumed, given the attainment of a plateau of clinical and functional improvement. However, it's important to note that the complete prognosis of this pathology is not yet fully understood [4,7,9].

Of note, the patient presently engages in activities akin to those pre-surgery, with her running being comparable and her jumping slightly less proficient than before. However, it is pertinent to acknowledge that prior to surgery, the patient experienced similar deficits in running and jumping abilities. While it is plausible that these deficits may have persisted for over 20 years, the extent to which the patient adapted to these limitations or was aware of them remains uncertain. Consequently, post-surgery, she does not perceive any notable changes in her daily functioning. Hitherto, there have been no discernible alterations in autonomy or activities of daily living relative to the pre-surgery period, with the patient expressing high satisfaction with the outcome.

## Conclusions

In conclusion, this case report sheds light on the diagnostic and management challenges associated with FAVA, emphasizing the necessity of a multidisciplinary approach. Surgical resection remains the treatment of choice for symptomatic lesions and progressive contractures, underscoring the importance of a comprehensive understanding of functional anatomy and post-surgical alterations for structuring effective rehabilitation programs. The implementation of an optimized rehabilitation program is crucial for maximizing functional capacity and participation in patients undergoing gastrocnemius resection. This case underscores the importance of addressing pain, contracture, loss of muscle mass, imbalance, and functional improvement as part of the patient approach, with multidisciplinary teamwork playing a pivotal role in achieving optimal outcomes. Furthermore, the challenges highlighted in diagnosing and managing FAVA emphasize the need for continued research and clinical discourse to enhance our understanding of this rare condition. Overall, the holistic care provided to this patient demonstrates the value of a collaborative and tailored approach to optimizing patient functional recovery and quality of life.
